# Expression and Immunogenicity Analysis of Recombinant *Leptospira Interrogans* Surface Protein LigA in Mouse Model

**DOI:** 10.1002/vms3.70360

**Published:** 2025-05-19

**Authors:** Aida Chalesh, Pejvak Khaki, Soheila Moradi Bidhendi, Majid Tebianian, Morteza Taghizadeh Tarnabi

**Affiliations:** ^1^ Department of Microbiology, North Tehran Branch Islamic Azad University Tehran Iran; ^2^ Department of Microbiology Razi Vaccine and Serum Research Institute Agricultural Research, Education and Extension Organization (AREEO) Karaj Iran; ^3^ Department of Immunology Razi Vaccine and Serum Research Institute Agricultural Research, Education and Extension Organization (AREEO) Karaj Iran; ^4^ Department of Medical Vaccine Razi Vaccine and Serum Research Institute Agricultural Research, Education and Extension Organization (AREEO) Karaj Iran

**Keywords:** immune system, *Leptospira interrogans*, Leptospirosis, Ni‐NTA affinity purification, recombinant Leptospiral immunoglobulin‐like surface protein A

## Abstract

**Background:**

Pathogenic strains of spirochetes of *Leptospira* spp. cause a globally distributed zoonotic disease called leptospirosis. The disease has several clinical manifestations, ranging from asymptomatic and subclinical infection to fatal and severe forms.

**Hypothesis/objectives:**

The aim of this study was to produce a recombinant Leptospiral immunoglobulin‐like surface protein‐A (r‐LigA) antigen of *Leptospira interrogans* in a prokaryotic expression system and to assess its efficacy in a mouse model.

**Materials and methods:**

The optimal epitopes of the LigA protein were identified via bioinformatics studies. The pET32a^+^‐LigA plasmid construct was cloned into *E. coli* Top10‐DH5α, expressed in *E. coli* pLysS strains, and subjected to different IPTG concentrations at different times and temperatures. The expressed r‐LigA was purified using nickel‐affinity (Ni‐NTA) chromatography from the insoluble fraction and reassessed by SDS‐PAGE, western blotting, dot blotting, and Bradford assay. Female Balb/C mice were immunised subcutaneously with r‐LigA alone or emulsified in Freund's adjuvant and subsequently boosted at 2 and 4 weeks. Specific antibody levels were evaluated by indirect ELISA.

**Results:**

Bioinformatics analysis identified the key antigenic region of LigA spanning amino acids 852 to 1210. Colony PCR and digestion confirmed the successful transformation. Induction using 0.5 mM IPTG at 30°C for 5 h was found to be optimal. Overexpression of r‐LigA under optimised conditions accumulated proteins as inclusion bodies. Purification of r‐LigA under native conditions using optimised Ni‐NTA yielded 1050 µg/mL protein and high immunogenicity by effectively stimulating the immune system in female Balb/C mice.

**Conclusions:**

These findings support r‐LigA as a strong candidate for future leptospirosis diagnostic tools and subunit vaccine development.

## Graphical abstract

Implemented strategies in designing, production, purification and immunogenicity of the recombinant Leptospiral immunoglobulin‐like surface protein‐A (r‐Lig A) as a vaccine candidate.

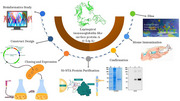

**Abbreviations**
6His: Hexa‐histidine
BID: Bacterial Ig‐like domain
Big_2: Bacterial Ig‐like group 2
BSA: Bovine serum albumin
CFA: Complete Freund's adjuvant
ELISA: Enzyme‐linked immunosorbent assay
FH: Factor H
FHL‐1: FH‐like 1
FHR‐1: FH‐related 1
IEDB: Immune Epitope Database
IFA: Incomplete Freund's adjuvant
IPTG: Isopropyl ß‐D‐1‐thiogalactopyranoside
LB: Luria–Bertani
NiNTA: Nickel nitrilotriacetate
PBS: Phosphate buffered saline
PMSF: Phenylmethanesulphonyl fluoride
PVDF: Polyvinylidene fluoride membrane
r‐LigA: Recombinant Leptospiral immunoglobulin‐like surface protein A
RT: Room temperature
RVSRI: Razi Vaccine and Serum Research Institute
TBST: Tris‐Buffered Saline‐Tween 20
TMB: 3,3′,5,5′‐Tetramethylbenzidine
TNB: 5‐thio‐2‐nitrobenzoic acid
TRX: Thioredoxin‐like moiety
TTBS: Tween‐20 in TBS

## 1 Introduction

Leptospirosis is a serious global zoonotic disease caused by pathogenic the serovars of *Leptospira* species (Calvopiña et al. 2023; Golab et al. 2020; Palaniappan et al. 2006; Sykes et al. 2011). Based on the carbohydrate component of the bacterial lipopolysaccharide, more than 250 serovars have been reported. These distinctive serovars with epidemiologic importance were adopted indifferent hosts. Resistance to leptospirosis is serogroup‐specific, and knowledge of the serogroup causing the disease in a specific geographic region is important for vaccine development (Sykes et al. 2011). The genus Leptospira has one species, *L. interrogans*, which is concurrently based on antigenic relatedness and classified into 27 serovars (sixteen pathogenic, five intermediate, and six saprophytic serogroups) in the CIS countries. Leptospirosis is prevalent worldwide, particularly in tropical, subtropical, and humid regions with inadequate sanitary conditions (Breda et al. 2015; Golab et al. 2021). Although the main reservoirs of these Gram‐negative bacteria are rodents, various mammals also harbour them (Inada et al. 1916). Humans as terminal hosts are infected via direct or indirect contact with urine or urine‐contaminated water or soil (Breda et al. 2015). During the early stages of the disease, approximately 90% of patients exhibit nonspecific symptoms, such as headache, fever, diarrhoea, vomiting, and joint pain. In some cases, patients may have flu‐like symptoms, respiratory distress, pulmonary haemorrhage, meningitis, and renal failure (Palaniappan et al. 2006; Breda et al. 2015; Bharti et al. 2003). In rare cases, acute respiratory failure with fatal pulmonary haemorrhage has been reported (Borer et al. 1999). As a result, it is challenging to differentiate clinical syndromes in humans.

Currently, there is no widely available vaccine against leptospirosis that provides heterologous protection in humans (Garba and Dirie 2022). Vaccine development is an initial and main strategy to combat the disease. Though the whole‐cell vaccine's effectiveness is limited by its short duration of immunity, differences in serovar specificity, and potential side effects, which can lead to a significant possibility of recurrence (Lin et al. 2016; Vedhagiri et al. 2009). Therefore, it is necessary to design and develop an efficient vaccine with lower levels of toxicity and greater immunity (Dellagostin et al. 2011). Studies have shown that the *L. interrogans* protein promotes cross‐protection against heterologous challenges (Palaniappan et al. 2006). Thus, most studies on recombinant Leptospiral vaccines have focused on the outer membrane proteins of spirochetes. Despite the identification of Leptospiral antigens such as OmpL1, LipL41, LipL36, LipL32, and LipL21, only a few studies have been conducted on their use in recombinant vaccine development (Golab et al. 2020; Palaniappan et al. 2006; Vedhagiri et al. 2009). Two Leptospiral immunoglobulin‐like surface proteins, A (LigA) and B (LigB), are related to pathogenic serovars that interact with multiple extracellular matrix components, such as collagen, fibrinogen, laminin, fibronectin, and elastin, which probably facilitate bacterial attachment to host tissues and colonisation (Breda et al. 2015; Koizumi and Watanabe 2004). Leptospira expressing LigA or LigB proteins exhibit stronger adhesion to the extracellular matrix and fibronectin in vitro (Palaniappan et al. 2002; Figueira et al. 2011; Kumar et al. 2023). On the other hand, Lig proteins help the immune escape of pathogenic Leptospira by binding to inhibitors of complement system factor H (FH), FH‐like 1 (FHL‐1), FH‐related 1 (FHR‐1), and C4b‐binding protein (Castiblanco‐Valencia et al. 2012). In 2002, the LigA (130 kDa) from *Leptospira interrogans* was discovered and found to elicit an immune response. LigA is a surface‐exposed lipoprotein that is highly immunogenic due to its unique structure. This structure is composed of 13 homologous tandem repeats of bacterial Ig‐like group 2 (Big_2) domains and a bacterial Ig‐like domain (BID), which is also the first to be expressed only during infection. These domains are homologous with the immunoglobulin‐like domain of *E. coli* intimin, the invasin of *Yersinia pestis* and a cell‐binding domain of *Clostridium acetobutylicum*. The non‐identical region of LigA form epitopes involved in the induction of the protective immune response (Dellagostin et al. 2011; Palaniappan et al. 2002; Wang et al. 2007). Domains 1'–7' are shared between LigA and LigB with no protection, whereas the domains 7'–13' regions are variable and unique to LigA, and an immune‐protective effect has been observed primarily in domains 10'–13' (Haake and Matsunaga 2021). According to previous studies, some LigA proteins may have value in sero‐diagnosis or could be a promising subunit vaccine candidate that provides high levels of protection in animal models against leptospirosis (Faisal et al. 2008; Silva et al. 2007).

A meta‐data analysis of published articles from Middle Eastern countries revealed that Icterohaemorrhagiae, Grippotyphosa, Sejroe, Canicola, Autumnalis and Pomona are the most prevalent Leptospira serogroups. These epidemiological and geographical heterogeneities in Middle Eastern countries emphasise that leptospirosis should be prioritised as a public health problem in this region and necessitate the introduction of an effective heterologous vaccine (Harran et al. 2022). On the other hand, several previous studies have proved that Immunisation with Lig proteins generates a strong humoral immune response. This response is characterised by a cross‐reaction between a homologous protein and a heterologous protein, as assessed by ELISA (Evangelista et al. 2017). As, variable regions of LigA are highly antigenic and exhibit an anti‐Lig antibody response during leptospirosis infection. The aim of this study was to design a molecular construct containing immunogenic epitopes that can be expressed and purified in a prokaryotic system. Furthermore, the immunogenicity of this recombinant protein should be investigated in an animal model to verify its efficacy.

## 2 Materials and Methods

### 2.1 Composition and Design of Chimeric R‐LigA Protein

Complete sequences of *Leptospira interrogans* as immuno‐dominant selection patterns were retrieved from the NCBI database (AC# WP_002093809.1) and stored in FASTA format. The LigA protein counterparts were separated into CDS. Subsequently, the selected protein sequence was analysed using the UniProt database (https://www.uniprot.org/). The partial CDS of highly conserved immuno‐dominant domains were subjected to the Immune Epitope Database (IEDB) (http://tools.iedb.org/mhci/) to identify optimal conserved amino acid regions that stimulate the immune system. in vivo half‐life, and stability indices were evaluated using the ProtParam tool (https://www.expasy.org/resources/protparam) (Wilkins et al. 1999).

### 2.2 Cloning and Expression Vector Synthesis

In this study, pET32a^+^ was used as the cloning vector of the construct. The construct was designed by insertion of the selected LigA sequence into the EcoRI and BamHI restriction sites in a multiple cloning site frame–with *a thioredoxin‐like moiety (TRX)* and *hexa‐histidine (6His) tag* sequence at the N‐terminal end (6xHis/Ni‐NTA)–carrying the ampicillin resistance gene. The r‐LigA‐pET32a^+^ plasmid was synthesised by General Biosystems (Anhui, USA).

### 2.3 Transformation of pET32a^+^‐LigA Into *E. coli* Top10‐DH5α and pLysS DE3

The recombinant plasmid pET32a^+^‐LigA was transferred to *E. coli* Top10‐DH5α cells using the conventional CaCl_2_ protocol to amplify the plasmid (Tang et al. 1994). Transformations were performed using using a standard heat shock protocol at 42^°^C and cultured on Luria–Bertani agar (LB) containing 50 µg/mL ampicillin. After 24 h of transformation, LB plates. Several single colonies were randomly selected and cultured on an antibiotic‐free medium to ensure the transformation of r‐LigA into *E. coli* via polymerase chain reaction using reverse T7 universal (Gene Link, Cat# 26‐3000‐13) and forward T7 universal (Gene Link, Cat# 26‐3000‐13) primers. Bacterial amplified plasmids were extracted using Roche kits (Cat. No. 11 754 777 001) according to the manufacturer's instructions.

Additionally, restriction enzyme digestion with EcoRI and BamHI was performed to determine the presence of insert DNA in the pET32a^+^‐LigA plasmid vector before downstream transformation (Liu and Yang 2012). To achieve enhanced and enhanced expression, extracted Plasmids (500 ng) were transformed into *E. coli* pLysS DE3 competent cells via the heat shock method and screened as described above.

### 2.4 Expression of Chimeric R‐LigA

To induce the overexpression of the chimeric r‐LigA protein in *E. coli* pLysS DE3, cells were grown overnight in 2× Yeast Extract Tryptone medium containing 50 µg/mL ampicillin at 37°C. Upon reaching OD_600_ up to 0.6–0.8, different concentrations (0.1, 0.3, and 0.5 mM) of Isopropyl ß‐D‐1‐thiogalactopyranoside (IPTG) were added and incubated for 3, 5, and 16 h at different temperatures (25°C, 30°C, and 37°C) while shaking at 220 rpm. The bacteria were then harvested via centrifugation (5500 rpm for 5 min at 4°C), and the pellets stored at –70°C for downstream experiments.

The pellets were re‐suspended in 10 mL of Phosphate Buffered Saline (PBS 1×) and sonicated (UP200 St, Hielscher Ultrasonics, Germany) 12 times for 1 min pulses with 1 min intervals while the samples were immersed in an ice bath. To inhibit protease activity, 10 µL of Phenylmethanesulphonyl Fluoride (PMSF) was added. The samples were then centrifuged at 15,000 rpm for 15 min at 4°C, supernatants and pellets were collected separately and then subjected to SDS‐PAGE to compare their solubility and yield.

### 2.5 Purification of the Chimeric R‐LigA Protein

In this study, 6xHis/Ni‐NTA affinity protein purification was performed as a fast and versatile technique. Sonicated pellet were purified using a Ni‐NTA resin column (Bio‐Rad, Hemel Hempstead Hertfordshire, UK) containing Ni‐NTA agarose (Qiagen, Hilden, Germany) as follow. The column was pre‐equilibrated by washing once with Elution Buffer (10 mM NaH_2_PO_4_, 190 mM Na_2_HPO_4_, 500 mM NaCl, 8 M urea, 500 mM Imidazole, pH 4.0). It was then washed twice with distilled water, followed by a final wash with Denaturing Binding Buffer (150 mM NaH_2_PO_4_, 50 mM Na_2_HPO_4_, 500 mM NaCl, 8 M urea, pH 7.8), followed by incubation for 1 h at room temperature (RT). The pellet proteins were mixed with Denaturing Binding Buffer, incubated for 10 min at RT, and then sonicated again as described above. Finally 20 µL PMSF added and centrifuged for 20 min at 5000 rpm and 4°C. The supernatant of each sample was loaded into the columns and shaken horizontally for approximately 45 min on ice with constant agitation (200 rpm) to enhance the interaction between the His‐tag tails of Ni‐NTA resins. The columns were then set vertically to allow the resin to settle before elution. The column was washed once with the Denaturing Binding Buffer, twice with Denaturing Washing Buffer (150 mM NaH_2_PO_4_, 50 mM Na_2_HPO_4_, 500 mM NaCl, 8 M urea, pH 6.0), and finally twice with Elution Buffer. All eluates were collected, pooled, filtered (0.45 µm membrane), and stored. The purity of the r‐LigA protein at each step was assessed using SDS‐PAGE (Gharakhani et al. 2023).

The most pure fraction of eluted r‐LigA protein was quantified using the conventional Bradford assay. Briefly, Bovine serum albumin (BSA) was used to generate a standard reference curve. 100 µL of protein solution was mixed with 100 µL of Bradford reagent dye (coomassie brilliant Blue G‐250), and after 20 min of incubation at RT, the absorbance was measured at 630 nm.

### 2.6 Immuno‐Blotting Analysis of the Chimeric R‐LigA Protein

To perform dot‐blot analysis, a polyvinylidene fluoride membrane (PVDF) (Hybond‐P, Amersham Biosciences, UK) was soaked in methanol and dried at RT. Then 2 µL of protein were immobilised onto the membrane and stored at RT. The membranes were then blocked with 5% BSA in Tris‐Buffered Saline‐Tween 20 (TBST) for 40 min at RT. Consequently, the membrane was washed in 1× TBST three times for 10 min, each with gentle rocking. The proteins were reacted with conjugated anti‐His tags antibodies (Thermo Fisher Scientific, USA) at RT for 1.5 h. Finally, after extensive washing with TBST once for 15 min and twice for 5 min, the reaction was visualised using 5‐thio‐2‐nitrobenzoic acid (TNB).

To perform western blot analysis, chimeric r‐LigA protein was transferred from the separation SDS‐PAGE gel to a PVDF membrane (Hybond‐P, Amersham Biosciences, UK) using a Mini Trans‐Blot system (Bio‐Rad, CA) according to the manufacturer's instructions. The proteins were blocked on the membrane by 0.1% BSA ‐TTBS (0.05% Tween‐20 in TBS) at RT for 40 min on a shaker, washed three times with 0.07% TTBS, and subjected to Leptospiral‐positive serum at RT for 2 h. The protein was reacted with horseradish peroxidase‐conjugated to anti‐6His ‐tag monoclonal antibody (Thermo Fisher Scientific, USA) at RT for 2 h. Finally, the reaction was visualised using TNB.

### 2.7 Immunisation of Mice to R‐LigA Protein

All procedures involving mice handling were approved by the Research Ethics Committees of Islamic Azad University‐ North Tehran Branch (approved IR.IAU.TNB.REC.1401.049). Twenty‐five female Balb/C mice aged 6–8 weeks were immunised. Briefly, animals were randomly divided into five groups, each consisting of 5 mice. The groups were injected subcutaneously on days 0 as follow; negative control (100 µL PBS), Leptospira vaccine control (RVSRI, Karaj, Iran), r‐LigA with Complete Freund's adjuvant (CFA) (RVSRI, Karaj, Iran) in a 1:1 ratio (100 µL r‐LigA‐adj), and r‐LigA (100 µL r‐LigAvar). The experiment was followed by a booster injection, as described above, with a difference in the adjuvant form, which was Incomplete Freund's Adjuvant (IFA), on days 14 and 28. Sera obtained in 6 steps with 10‐day intervals (0–50 days), and their titres were determined by ELISA.

### 2.8 Enzyme‐Linked Immunosorbent Assay (ELISA)

Optimal dilutions of the antigen and antibody were determined after performing the checkerboard test and were used to perform the ELISA tests. The purified protein was prepared at a concentration of 5 mg/mL in Coating Buffer (50 mM carbonate buffer: Na_2_CO_3_/NaHCO_3_, pH 9.6). Polystyrene plates (SPL Life Sciences, Pocheon, Korea) were filled with 100 µL of chimeric r‐LigA protein for 16 h at 4°C. The wells were then washed three times with 300 µL of Wash Buffer (PBS+ 0.05% Tween 80: PBST) using an ELISA washer (BioTek, ELX50, USA). Blocking was performed with 300 µL of 3% skim milk solution for 1 h at 37°C. Then, 100 µL of sera (diluted 1:50 in 1% skim milk solution) were added to the wells and incubated for 1 h at 37°C. Total IgG and IgM against chimeric r‐LigA were evaluated using rabbit anti‐mouse horseradish peroxidase antibody (1:15,000). After 1 h of incubation at 37°C, the wells were washed three times with Wash Buffer. The 3,3′,5,5′‐Tetramethylbenzidine (TMB) substrate was added to each well, and after 15 min, the reaction was stopped by adding a stop solution (sulphuric acid 10%). The plates were read at 450 nm using an ELISA reader (Awareness, Stat Fax, 2100, USA), and data were analysed.

### 2.9 Statistical Analysis

One‐way analysis of variance (ANOVA) and *t*‐tests were used to compare immune responses between treatment groups. For all tests, statistical significance was set to *p* ≤ 0.05. All analyses were performed using GraphPad Prism 5 (GraphPad Software, Inc., La Jolla, CA).

## 3 Results

### 3.1 Composition and Design of Chimeric R‐LigA Protein

In applied immune‐informatics approach the positions 852–937 (ligA9: BID_2: ELTEIVLNPTSSHKAKG LTENFKATGVFTDNSTKDITDQVTWKSSNAAYAKISNATGSKGVVNALSKGTSHISATLGSISSANATF), 943–1029 (ligA10: Big_2: RIASIEVTPNNFFLIKGLSHPFKAIGIYTDNTKTDITKQVSWSSSDPNVAS IDNTFSLAGSATAIDDGKTNITATLSDSMSASTTLY), 1034–1119 (ligA11: Big_2: VLIDIEVKPSIFVLSE GLTLQLTATGIYSDHSTHDLTQVVQWTSSKPSNIAIENTAGKK GKVTALAFGDSEFTATYDSIKSNRA WI), and 1125–1210 (ligA12: Big_2: KLVNITIS SSQVLTDKGSAQQFKAIGTFQGGSQLDLTDLVTWKS SDSKVVSISNSNDDRGLTTALSVGSSKISAIYGSIHSDSIDF) amino acid residues contained highly conserved antigenic residues. For proper conformation, selected domains connected to adjacent Ig‐like domains by linkers as follows: (1) (QVTPA), (2) (VTSA), and (3) (FVNDE).

The IEDB Bepipred predicted 39 different overlaps and had a positive IEDB immunogenicity score.

The ProtParam results representing the physicochemical properties of the designed chimeric r‐LigA protein are presented in Table 1. The instability index of the designed chimeric protein was 24.21, implying that the protein is stable (more than 10 h) in *E. coli*.

**TABLE 1:** Physicochemical properties of protein sequences based on the ProtParam results.

Parameters	
Number of amino acids	359
Molecular weight	37897.12 = ˷38 kDa
Theoretical pI	5.69
Instability Index	24.21
Aliphatic Index	85.88
Grand average Of hydropathicity	−0.114
Estimated half‐life	1 h (mammalian reticulocytes, in vitro) 30 min (yeast, in vivo) >10 h (*Escherichia coli*, in vivo)

The identified aliphatic index data indicate stability over a wide temperature range, whereas the negative GRAVY (–0.114) indicates the hydrophilic and soluble nature of the protein.

### 3.2 Transformation of pET32a^+^‐LigA Into *E. coli* Top10‐DH5α and pLysS DE3 Cells

**FIGURE 1:**
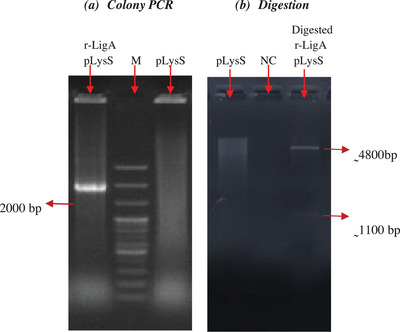
Transformation confirmation electrophoresis on 1% agarose gel (M: Marker, NC: Negative control).

Both of the *E. coli* Top10‐DH5α and pLysS DE3 cells were transformed with pET‐32a^+^‐LigA and yielded several colonies successfully in the presence of ampicillin. The presence of the target gene in plasmids and susceptible cells was confirmed in multiple random clones using PCR. Colony PCR using T7 universal primers resulted in a 2000‐bp band amplification (Figure 1a). Furthermore, digestion of the purified recombinant plasmid of the transformed cell by EcoRI and BamHI yielded two fragments with a plasmid size of 5.9 kb, whereas the size of the *r‐LigA* gene was 1085 bp, as expected (Figure 1b). These findings confirmed the incorporation of the inserted *r‐LigA* gene into the plasmid and successful transformation.

### 3.3 Expression, Purification and Immunoblotting Analysis of Chimeric R‐LigA Protein

**FIGURE 2:**
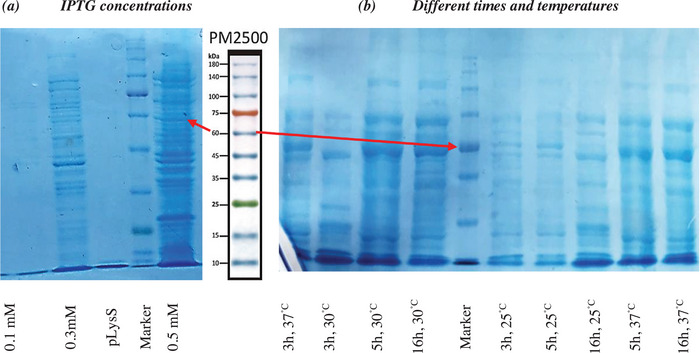
SDS‐PAGE of expressed r‐LigA in *E. coli* (pLysS) under different conditions (Marker: PM2500).

**FIGURE 3:**
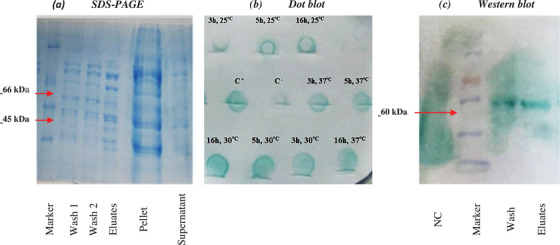
(a) SDS‐PAGE result after Ni‐NTA purification (Marker: PM2500. (b) Dot blot results comparing temperatures of 25°C and 30°C at 5 and 16 h. (c) Western blot after Ni‐NTA purification. (NC: Negative Control, Marker: PM2500).

To examine the effects of different IPTG concentrations, times, and temperatures on the production of r‐LigA, the relative level of expression was analysed using SDS‐PAGE. The yield of purified r‐LigA protein (1050 µg/mL) was higher than previous reports using alternative expression systems, indicating an optimised purification process. pET32a^+^vector contains 109 amino acid Trx•Tag thioredoxin protein, which produces an approximately 18.5‐kDa peptide. Transformed *E. coli* pLysS DE3 cells successfully expressed r‐LigA using various concentrations of IPTG, as indicated by a band of approximately 56.5‐kDa (pET32a^+^; ˷18.5‐kDa +LigA; ˷38‐kDa) compared with the lack of IPTG as a control (pLysS; Lane 1 Figure 2a). The best conditions for the high‐level r‐LigA protein expression were achieved at 0.5 mM IPTG, at 30°C, although in case of the time both 5 and 16 h yield were optimum. Other IPTG concentrations and temperature also demonstrated sufficient results confirmed by SDS‐PAGE and dot blot analysis as well (Figures 2a, b, and 3a, b).

The results of post‐sonication SDS‐PAGE analysis indicated that the protein content in the pellets was significantly higher than the supernatant, which explains the insoluble form of aggregated proteins in inclusion bodies. Protein purification using the Ni‐NTA resin column also produced a sharp band between 66 and 45 kDa, attributed to r‐LigA. It is noteworthy that the washing steps caused non‐specific proteins to be released from the resin (Figure 3a). Western blotting confirmed a nearly 58‐kDa distinct band (Figure 3c).

### 3.4 Enzyme‐Linked Immunosorbent Assay (ELISA)

**FIGURE 4:**
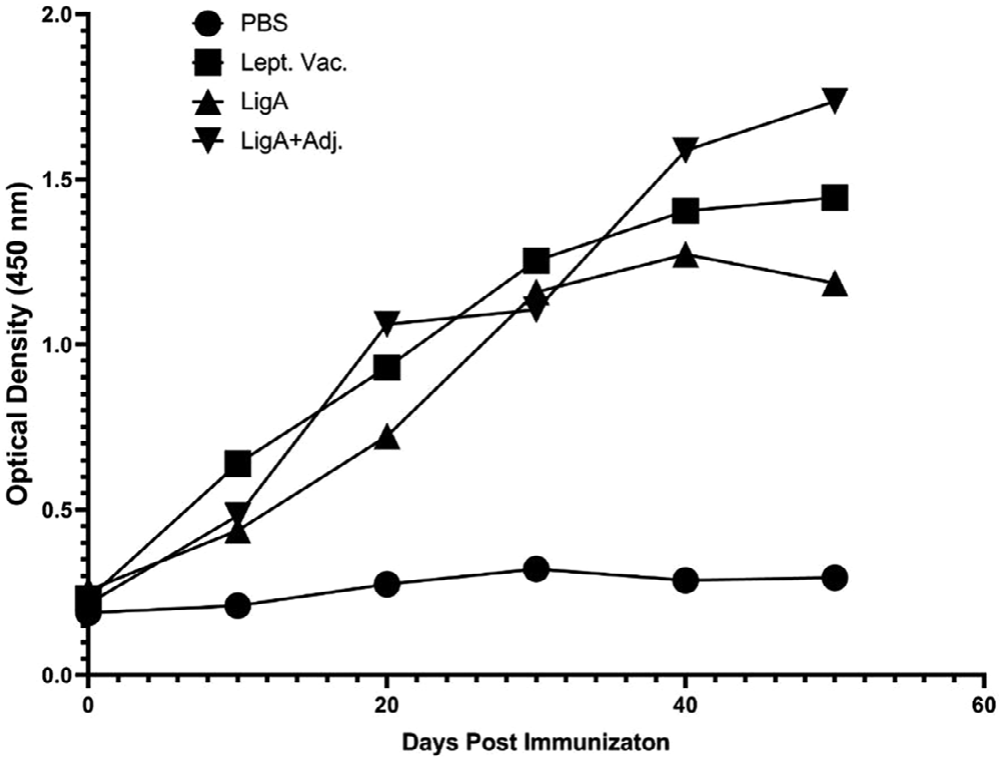
Kinetics of Antibody response against LigA Antigen in different immunised animals.

**FIGURE 5:**
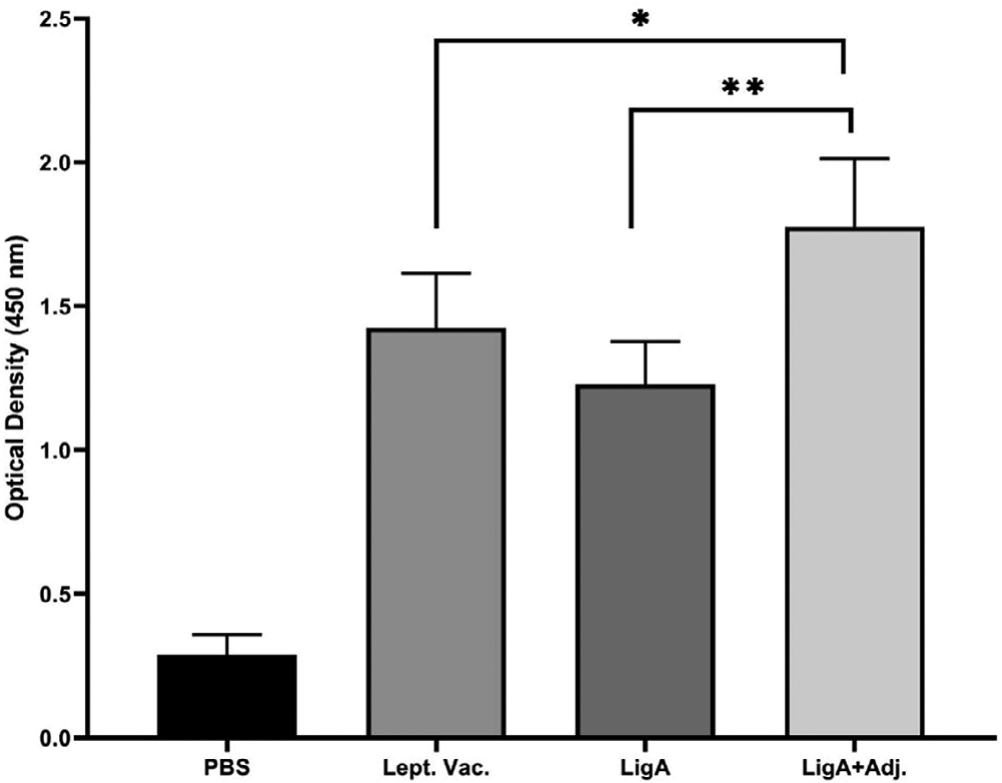
Comparison of anti‐LigA antibody response across different test groups in the last day of study (day 50). Results represent mean and SD of *n* = 5, *p < 0.05; **p < 0.001.

Antigenicity of the purified r‐LigA protein evaluated by western blotting revealed that r‐LigA at 1:2000 dilution interacts with Leptospiral‐positive serum. The levels of specific antibodies against the chimeric r‐LigA protein were determined using indirect ELISA. According to the kinetic results of humoral immune responses (Figure 4), the animals that received the Leptospiral Vaccine exhibited a gradual increase in anti‐LigA antibody levels over time. This indicates that a moderate immune response was induced by the vaccine. Similarly, the administration of recombinant LigA (r‐LigA) elicited a significant immune response. However, the immune response from r‐LigA was not as robust as that observed in the r‐LigA+Adj group. Notably, the administration of recombinant LigA with adjuvant (r‐LigA+Adj) resulted in the highest optical density, peaking on day 40. This indicates that the addition of an adjuvant significantly enhanced the immune response, yielding the strongest reaction among all groups tested. A statistical comparison of specific anti‐LigA antibody levels measured on the final day of the study showed that the LigA+Adj formulation elicited the strongest immune response among the tested groups. This was evident from a significantly higher OD compared to both the Leptospira vaccine and r‐LigA without adjuvant (*p* < 0.05 and *p* < 0.001 respectively) (Figure 5). The Leptospira vaccine group exhibited a moderate response, whereas the r‐LigA (without Adj) group showed a substantial but lesser response compared with the adjuvant‐enhanced formulation.

## 4 Discussion

Although commercial Leptospira vaccines for animals are available, few are approved for human use because of short‐term immunity, limited cross‐protection against different serovars, and potential side effects (Dellagostin et al. 2011; Faisal et al. 2008; Barazzone et al. 2022). In contrast, inactivated whole cells (bacteirn) vaccines are the most commercially available vaccine, primarily for livestock and domestic animals use for over five decades. Although protective against lethal infection, these vaccines had several shortcomings. The main issues were that they elicited protection against only a narrow range of serovar, lacked consistency and repeatability in production, may not have been effective in preventing disease transmission, caused reactogenicity, were high in production costs, and failed to induce long‐term immunological memory. Moreover, they do not promote long‐term protection, where several booster doses are necessary (Dellagostin et al. 2011; Barazzone et al. 2022; Bashiru and Bahaman 2018). Recombinant protein vaccines, as a member of subunit vaccines class, have been identified as promising candidates due to their avirulent nature, reduced bio‐hazardous potential, non‐infectious and non‐viable properties, and well‐defined composition (Bashiru and Bahaman 2018). On the other hand, it is well known that Leptospira surface proteins frequently exhibit cross‐reactivity, whereas the surface lipopolysaccharide (LPS) components are predominantly serovar‐specific, eliciting antibodies that are specific to those serovars (Boonciew et al. 2024). Therefore, to provide an efficient strategic approach to introduce a new vaccine candidate, the objective of this study was set to design a construct of recombinant LigA protein followed by transformation, expression, and purification in the prokaryotic system, along with the evaluation of its immunogenicity in mice.

In this study, based on the multiple alignment interspecies, the similarity at both levels of identity of the deduced ligA nucleotide and amino acid sequences ranged from 91.9% to 99.7% among 82 *L. interrogans* and three *L. kirschneri* pathogenic serovars. The sequence identity of LigA was relatively higher in *L. interrogans* than in *L. kirschneri* serovars. These findings are in congruence with the results of McBride et al. (2009), who conferred a 70%–99% identity in *lig* genes and 68%–99% interspecies identity in conserved regions within the *lig* genes, whereas 89% mean identity was observed in LigA Big domains 1–10 and 80% in full‐length LigA. Of note, the C‐terminus of LigA (Big domain 11–13) exhibited the highest variability.

It has also been reported that LigA is present only in pathogenic *L. interrogans* and *L. kirschneri* strains, and 80%–97% homology is predicted for the amino acid sequences of the LigA protein in these serovars (Koizumi and Watanabe 2004; McBride et al. 2009). Because it has been reported that the entire LigA open reading frame (with/without signal peptide) expression in *E. coli* is very low and leads to toxicity (Palaniappan et al. 2006), the conserved and variable regions of LigA were used in this study. Construct of r‐LigA protein consisting of ligA9, 10, 11, and 12 domains inserted in the pET32a^+^ cloning vector correctly transformed into the *E. coli* Top10‐DH5α and pLysS DE3 cells. It has been suggested that the immune protection of the LigA protein construct should consist of at least three Big domains in which ligA11‐ and ligA12‐specific Big domains must be included. The probable mechanism of LigA‐mediated immune protection is related to its ability to adhere to extracellular matrix proteins such as fibronectin, collagen, and laminin in host cells thorough the 7–13 domains of LigA (Coutinho et al. 2011). There are some reports of the failure of homologous immunisation of denatured LigA7‐13 domains against lethal infection by the *L. interrogans* serovar Manilae strain L495, which is in contrast to previously successful reports involving *L. interrogans* serovars Manilae, Copenhageni, and Pomona (Koizumi and Watanabe 2004; Coutinho et al. 2011; Lucas et al. 2011). Alongside the differences in the strains and adjuvants used, the failure of immune protection probably indicates that the protective epitope is conformational rather than linear (Forster et al. 2013). According to previous reports, in this study, LigA11 and LigA12 incorporated in the construct facilitate proper conformational folding. Kumar et al. (2021) discovered that the LigA11 domain is the most immuno‐dominant domain involved in obtaining the complement regulator FH, and it binds to the host protease plasminogen. Moreover, the authors found that LigA∆11 is involved in TLR4 signalling, which results in macrophage maturation, in addition to MAPK signalling involving the MyD88 Adapter, which induces immune activation. These findings confirm the direct roles of the immune response in innate and adaptive immune responses (Kumar et al. 2021).

The *E. coli* expression system has a clear genotypic background, high gene expression levels, a short cultivation cycle, and verifiable tagged proteins, making it an excellent candidate for expression systems (Fu et al. 2015). As a part of this study, the r‐LigA construct transferred into *E. coli* Top10‐DH5α and pLysS DE3. In a study conducted by Silva et al. (2007), a 63‐kDa recombinant protein (corresponding to nucleotides 1873–3675 of the ligA) was cloned in pET100‐top5 and transformed to *E. coli* BL21 (DE3), induced protective immunity in a hamster model of leptospirosis (Silva et al. 2007). Interestingly, Palaniappan and McDonough (2002) failed to express r‐LigA in a pET system with a His‐tag fusion protein, whereas their efforts in the pGEX4T‐2 system were successful (Palaniappan et al. 2002).

Factors such as the promoter system, host‐vector compatibility, the concentration and toxicity of the recombinant product, IPTG concentration and its induction in proportional OD_600_, incubation time, and temperature on the expression of some products have been previously reported (Palaniappan et al. 2006). It is well documented that, unlike lactose and other galactosides, high concentrations of IPTG are not metabolised by cells and may have toxic effects and lower expression levels (Larentis et al. 2014). On the other hand, untested predetermined IPTG concentrations can reduce the cell and protein yields under non‐optimal conditions. The experimental data of the present study indicate that the maximum r‐LigA protein expression was obtained in the presence of 0.5 mM IPTG at 5 and 16 h post‐induction incubation at 30°C. Overexpression of plasmid‐encoded genes in recombinant bacteria triggers the transcription of heat shock genes and stress responses, resulting in protein aggregation into inclusion bodies (Villaverde and Mar Carrió 2003). Similar to previous studies, most r‐LigA was expressed in the insoluble fraction, but incubation at a higher temperature (30°C) may have caused inclusion body formation in this study. In a previous investigation conducted by Silva et al., in 2007, optimum conditions for LigA production in the *E. coli* BL21 (DE3) Star transformant were achieved at 37°C and 1 mM final IPTG (Silva et al. 2007). In an effort conducted by Rizkia et al., an IPTG concentration of 0.01 mM and reduction of the temperature down to 12°C resulted in a transcription and translation balance and minimised the formation of inclusion bodies in the *E. coli* ER2566 (pTWIN1‐pretrmbin‐2) expression system (Rizkia et al. 2015). Larentis et al. also reported the expression of soluble recombinant LigB (131–645 aa, 54 kDa) protein using pAE in *E. coli* BL21 (DE3) Star, and its induction using 0.1 mM IPTG at 28°C for 4 h, reaching higher cell densities and yielding 970 mg/L protein (Larentis et al. 2014).

It merits consideration that the expression of recombinant proteins in the form of inclusion bodies increases protein yield, in addition to facilitating the purification process (Seyedinkhorasani et al. 2020).

Generally, depending on the nature, size, physicochemical properties, affinity, and biological activities, one or more chromatographic steps are required for protein purification. The presence of His‐tag residues is the key factor of efficient Ni‐NTA affinity chromatography purification. Ni‐NTA, as a chelating adsorbent resin, binds tightly and efficiently to proteins (even those that are insoluble under native conditions or minor expressed ones), facilitating the purification of proteins in just one step (Gharakhani et al. 2023; Crowe et al. 1996). Once it was established that r‐LigA was present in the insoluble fraction, the inclusion bodies were sonicated and dissolved in the Denaturing Binding Buffer, and applied to a Ni–NTA column that had been pre‐equilibrated with the same buffer. This allowed r‐LigA to renature and bind to the column via the His‐tag. Meanwhile, the two washing steps eliminated nonspecifically bound proteins, and the Elution Buffer (containing imidazole) eluted strongly bound r‐LigA.

Given the outcome of the Bradford assay, the purified protein yield using this method was 1050 mg/L. A related study conducted by Hartwig et al. (2010) expressed rLigANI (61 kDa) in the eukaryotic expression system of *Pichia pastoris*, resulted in a significantly lower protein yield. Protein was purified using different methods and gained 70, 183, and 239 mg/L by ammonium sulphate precipitation, ultrafiltration, and lyophilisation, respectively. They postulated that the recombinant proteins were glycosylated by *P. pastoris*. This highlights the superiority of the prokaryotic expression system in terms of higher protein expression levels and the absence of de‐glycosylation challenges (Hartwig et al. 2010).

To date, besides aluminium hydroxide (alhydrogel) and Freund's adjuvants, other adjuvants like flagellin, CpGs, nanostructures, liposomes, and xanthan have also been widely studied. Despite its high reactogenicity in humans, Freund's adjuvant is the most potent commercially available option adjuvants for experimental antibody production (Grassmann et al. 2017; Harold and Stils 2005). According to reports, FCA injections are associated with a diverse range of injuries, from localised granulomas at the injection site to distant granulomas in the lungs, liver, and kidneys, necrotising dermatitis, and spinal cord compression from the injection site granulomas (Harold and Stils 2005).

In this study, each group of mice was primed and boosted with r‐LigA alone or along Freund's adjuvant. According to our results, the rLigA+Adj group elicited the most robust immune response, as evidenced by the highest optical density. Conversely, the r‐LigA without adjuvant also demonstrated a substantial immune response, albeit less pronounced than the LigA+Adj group. Similarly, the Leptospira Vaccine group exhibited a moderate immune response. In contrast, the PBS group did not display any significant immune response. These findings imply that incorporating an adjuvant into the r‐LigA antigen formulation significantly amplifies the immune response.

These results were in agreement with the findings of other researchers who showed better immunisation of LigA (amino acid residues 852 to 1107) when Freund's adjuvant was used (Silva et al. 2007; Coutinho et al. 2011). Leptospira surface proteins in combination with adjuvants like Freund's, liposomes, PLGA microparticles, xanthan gums, AddaVax, and Emulsigen‐D have varying degrees of protection, ranging from 50% to 70% (Varma et al. 2023). The results of this study indicated that Freund's adjuvant in combination with r‐LigA had significant effect on immune system stimulation. A study by Coutinho et al. (2011) demonstrated that when evaluating purified recombinant LigA protein (Ig‐like domains 10–13) along with Freund's adjuvant induce 100% protection in hamsters, sterilising immunity was not observed (Coutinho et al. 2011). The variability in the efficacy of Freund's adjuvant among the research groups may be attributed to differences in the experimental protocols used. A comparative study of the r‐LigA antigen using different adjuvants, particularly clinical adjuvants such as Montanide, MF59, and Adjuvant Systems (AS03, AS04), could provide valuable insights into its immunological mechanism. These adjuvants have shown strong potential in enhancing antigen‐specific immune responses and protective efficacy. Subunit antibodies are considered as a suitable candidates as can be combined with variety of adjuvants (de Oliveira et al. 2023).  Hence, to optimise the efficiency of r‐LigA, further research on different adjuvants is recommended.

In this study, *E. coli* was used as a preferred heterologous subunit vaccines expression platform, due to the broad range of available protocols, handling feasibility, and inexpensive production of this system. In conclusion, these findings highlight the successful expression and purification of r‐LigA using a bacterial expression system. Based on the results of the bioinformatics analysis, it can be said that the designed structure of the target gene has a favourable overlap with all leptospirosis serovars, which can be used in the future diagnostic methods of leptospirosis or recombinant vaccine. The development of recombinant leptospirosis vaccines is a complex process involving several challenges. Key challenges that should be addressed in further vaccine studies include determining the optimal formulation, demonstrating effectiveness in controlled challenges, achieving complete renal clearance, and consistently reproducing protective effects in different scenarios.

## Author Contributions

**Aida Chalesh**: Methodology; Investigation; Formal analysis; Visualisation; Writing–original draft. **Pejvak Khaki**: Supervision; Conceptualisation; Project administration; Resources; Funding acquisition, Methodology; Investigation; Validation; Writing–review & editing. **Soheila Moradi Bidhendi**: Supervision; Conceptualisation; Investigation; Resources; Project administration; Funding acquisition, Methodology; Validation; Writing—review & editing. **Majid Tebianian and Morteza Taghizadeh Tarnabi**: Data curation; Formal analysis; Software, Writing–review & editing. All authors read and approved the final manuscript.

## Ethics Statement

All the procedures for animal experiments were approved by the Research Ethics Committees of Islamic Azad University‐ North Tehran Branch, under IR.IAU.TNB.REC.1401.049 Approval ID and performed in accordance to the ethical principles and the national norms and standards for conducting Medical Research in Iran guidelines.

## Conflicts of Interest

The authors declare that they have no known conflicts of interest or personal relationships that could have appeared to influence the work reported in this paper.

### Peer Review

The peer review history for this article is available at https://www.webofscience.com/api/gateway/wos/peer‐review/10.1002/vms3.70360.

## Data Availability

The datasets containing all data analysed and supporting the results of this study will be available upon request from the corresponding author.
