# Correction to “Expression and Immunogenicity Analysis of Recombinant *Leptospira interrogans* Surface Protein LigA in Mouse Model”

**DOI:** 10.1002/vms3.70734

**Published:** 2025-12-23

Chalesh A., P. Khaki, S. Moradi Bidhendi, M. Tebianian, and M. Taghizadeh Tarnabi. “Expression and Immunogenicity Analysis of Recombinant Leptospira Interrogans Surface Protein LigA in Mouse Model.” *Veterinary Medicine and Science* 11, no. 3 (2025): e70360. https://doi.org/10.1002/vms3.70360.

In the Author Affiliations section, the second affiliation of Dr Soheila Moradi Bidhendi was omitted. The correct affiliation should include: Department of Microbiology, Razi Vaccine and Serum Research Institute, Agricultural Research, Education and Extension Organization (AREEO), Karaj, Iran.

We apologize for this error.

